# Evaluation of postoperative changes in condylar positions after orthognathic surgery using balanced orthognathic surgery system

**DOI:** 10.1186/s40902-022-00341-x

**Published:** 2022-03-17

**Authors:** Yong-Chan Lee, Hong-Bum Sohn, Young-Wook Park, Ji-Hyeon Oh

**Affiliations:** 1 Bestian Oral & Maxillofacial Surgery Clinic, 429, Dogok-ro, Gangnam-gu, Seoul, 06208 Republic of Korea; 2 Department of Orthodontics, Eton Dental Clinics, 98, Bangsong-gil, Chuncheon, Gangwondo 24364 Republic of Korea; 3 Department of Oral and Maxillofacial Surgery, College of Dentistry, Gangneung-Wonju National University, 7, Jukheon-gil, Gangneung, Gangwondo 28644 Republic of Korea

**Keywords:** Orthognathic surgery, Condylar position, Accuracy, Virtual surgical planning

## Abstract

**Background:**

Many studies on maintaining the condyle in a normal or anatomical position during orthognathic surgery have been conducted to stabilize surgical outcomes and prevent iatrogenic temporomandibular joint complications. The aim of this study is to evaluate the changes in condylar positions after orthognathic surgery using virtual surgical planning via the balanced orthognathic surgery (BOS) system.

**Methods:**

Postoperative changes in condylar position were retrospectively evaluated in 22 condyles of 11 patients with skeletal class III malocclusion who underwent orthognathic surgery using virtual surgical planning via the BOS system. The center point coordinates of the condylar head before and after orthognathic surgery were analyzed using voxel-based registration.

**Results:**

Changes in the condylar position mainly occurred downward in the y-axis (−1.09 ± 0.62 mm) (*P* < 0.05). The change in the x-axis (0.02 ± 0.68 mm) and z-axis (0.01 ± 0.48 mm) showed no significant difference between before and after orthognathic surgery.

**Conclusion:**

These results indicate that the changes in the condylar positions after orthognathic surgery using virtual surgical planning via the BOS system mainly occurred downward in the y-axis, with slight changes in the x- and z-axes. The change in the condylar position after orthognathic surgery using the BOS system is clinically acceptable.

## Background

Orthognathic surgical planning has improved with the development of computer-aided design/computer-aided manufacturing (CAD/CAM) technology [1–4]. Virtual surgical planning and rapid prototyping (RP) technology simulate various surgical plans and predict their outcomes using three-dimensional (3D) data for the dental arch and surrounding skeletal structures [1, 5, 6]. In orthognathic surgery, the planning time for virtual surgical planning is shorter than that for conventional surgical planning [7, 8]. Three-dimensional printed splints and guiding templates are used to transform virtual surgical planning to actual results [2, 9–11].

After orthognathic surgery, the condylar position can be changed by several factors, such as the fixation method, surgeon’s experience, and positioning of the proximal and distal segments of the mandible [12–14]. Maintaining the condyle in a normal or preoperative anatomical position after orthognathic surgery is critical to achieve a stable skeletal and occlusal outcome and prevent iatrogenic temporomandibular joint complications [15–17]. If the condyle is distracted from the glenoid fossa during surgery, immediate relapse may occur, whereas if it is located posteriorly, condylar resorption or late relapse may occur [18, 19].

However, there is a lack of consensus on the accuracy assessment of methods for evaluating the change in the condylar position [20]. Because there is no standardized method to measure postoperative changes, it is difficult to compare data from multiple studies and assess the effectiveness of new techniques [21]. In addition, if the reference point is manually reidentified, an error of 1 mm or more can be included every four repeated measurements, so there is a limit to the repeatability of 3D measurement in orthognathic surgery [22].

A condylar positioning device (CPD) was first introduced by Leonard in 1976 [23]. CPDs, developed by clinicians, are devices that precisely position the condyle during orthognathic surgery [19, 23]. The balanced orthognathic surgery (BOS) system was first introduced in 2015 as computer-assisted simulation surgery [24]. In the BOS system, a surgical wafer that functions as a CPD is manufactured with CAD/CAM and used for orthognathic surgery. The BOS system consists of four phases: planning and simulation, modeling, surgical, and evaluation. During the planning and simulation phase, a 3D model is established by merging the dentition scan image of the stone model and the computed tomography (CT) image of the skull, and orthognathic surgery is simulated using the BOS equation. During the modeling phase, a surgical wafer is manufactured using the RP machine. In addition, a cutting guide is prepared from the 3D RP model before surgery, and the miniplates are pre-bent from the 3D RP model operated as planned. During the surgical phase, orthognathic surgery is performed using these surgical tools. Finally, in the evaluation phase, virtual surgery and postoperative CT images are merged, and the error is analyzed.

The purpose of this study was to evaluate the changes in the condylar position after orthognathic surgery using virtual surgical planning via the BOS system.

## Materials and methods

### Sample patients

The Institutional Review Board of Gangneung-Wonju National University approved this retrospective study of patients, who were requested to produce a surgical guide with the BOS system for orthognathic surgery (GWNUIRB-R2021-64). The Institute of BOS provided retrospective anonymous data for 22 condyles of 11 patients (four men and seven women; mean age, 21.1 years; age range, 18–29 years) with skeletal class III malocclusion; the patients underwent orthognathic surgery using the BOS system. Four patients underwent only mandibular surgery, and seven underwent bimaxillary surgery. The average mandibular setback was 8.97 mm.

### Virtual surgical planning and surgical procedure

**Fig. 1:**
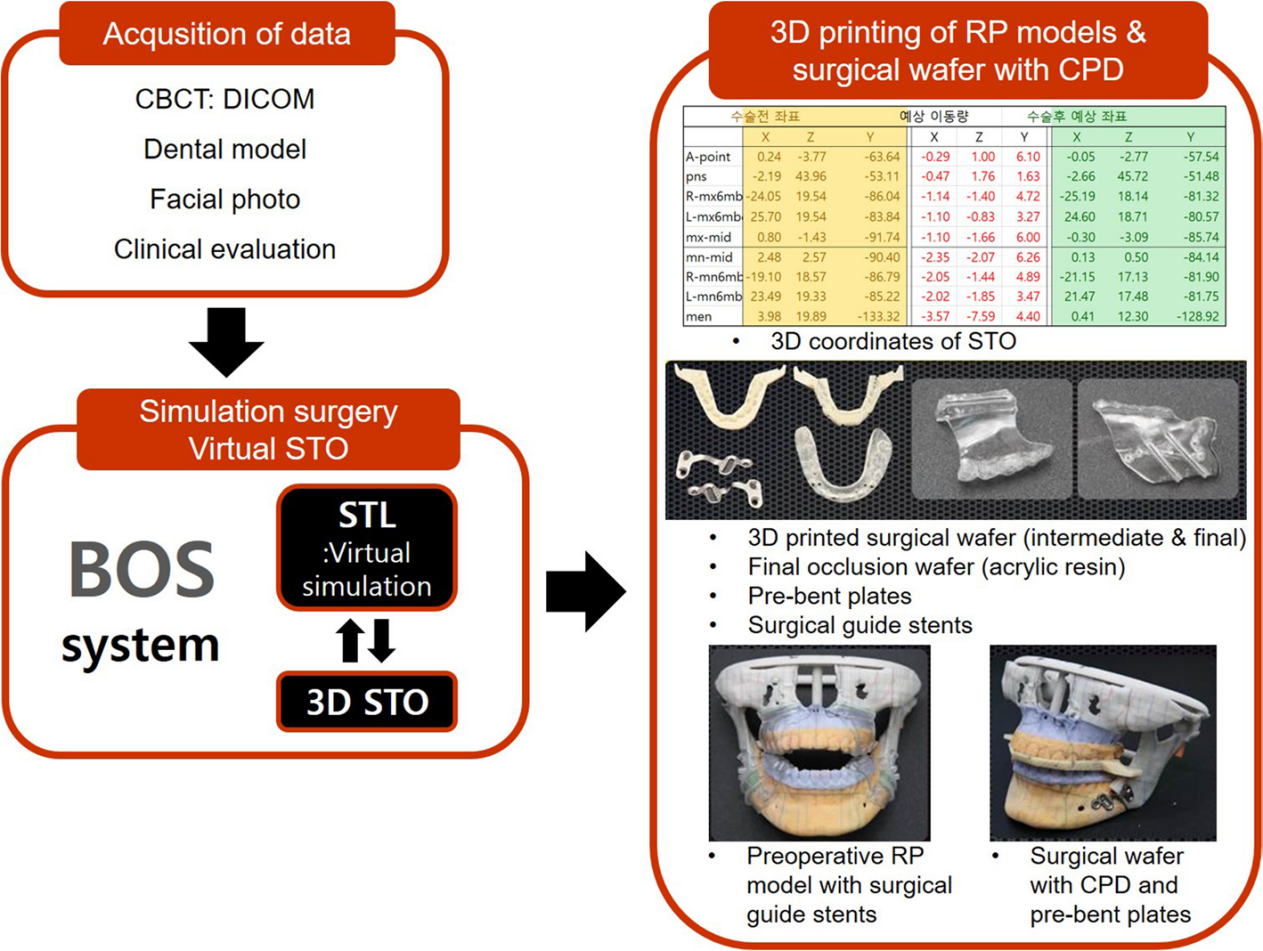
Preparation process for orthognathic surgery using BOS system

Virtual surgery was planned using the BOS system (Fig. 1). The cutting guide was manually produced in the RP model, and the wafer was manufactured using CAD/CAM. Bilateral sagittal split ramus osteotomy with or without LeFort I osteotomy was performed using the conventional method, and metal plates pre-bent from the RP model were used for fixation.

### Evaluation of surgical accuracy in BOS system

**Fig. 2:**
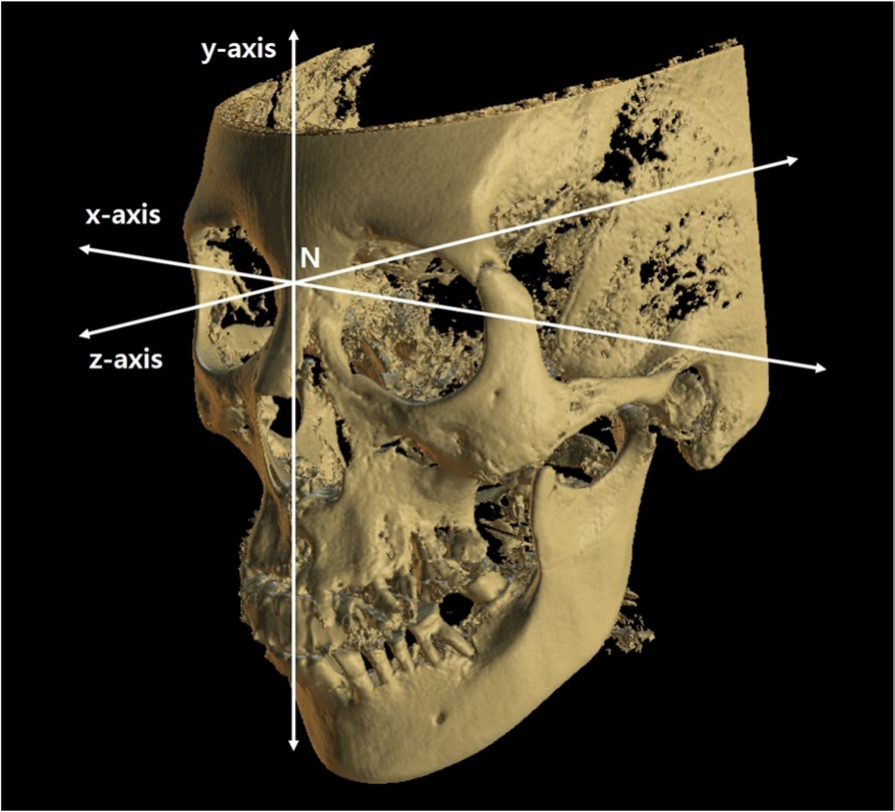
Three-dimensional coordinate system. The Frankfort horizontal plane is the reference plane, and the nasion is the center of all axes. The medial-lateral movement was evaluated by the x-axis. The vertical movement was evaluated by the y-axis. The anterior-posterior movement was evaluated by the z-axis

The nasion was set to the coordinate points (0, 0, 0), and the orientation was set to the Frankfurt horizontal (FH) plane. The x-, y-, and z-axes were used to set the coordinates (Fig. 2). The x-axis was a straight line parallel to the line passing through both orbitales on the FH plane. The y-axis was a straight line passing through the nasion and perpendicular to the FH plane. The z-axis was an anteroposterior line, with a straight line passing through the nasion parallel to the FH plane and perpendicular to the x-axis. The coordinates of the center points of the condyle heads on both sides were obtained. The center point of the condylar head was defined as the middle part between the lateral and medial poles. The center point coordinates of the condylar head before and after orthognathic surgery were compared. Postoperative CT was performed on postoperative days 0–3. The data for patients that underwent orthognathic surgery using the BOS system were analyzed after superimposing using voxel-based registration (Invivo5; Anatomage Inc., CA, USA). The quaternion was obtained by superimposing the CT data before and after surgery and converted to Euler’s angle, which was then calculated using the direction cosine matrix method to obtain the amount of change at each point. By substituting the coordinate values resulting from the designating points in the preoperative CT, the coordinate values in the postoperative CT were obtained.

### Statistical analysis

Statistical analysis was performed on the amount of changes in the coordinate values of each of the x-axis, y-axis, and z-axis before and after surgery in 22 condyles. After testing for normality using Kolmogorov-Smirnov test and Shapiro-Wilk test, statistical analysis was performed using the paired *t*-test, and the significance level was set at 0.05.

## Results

Table 1 lists the changes in coordinate values of each patient's preoperative and postoperative condylar heads. The changes in the condylar position were mainly observed downward on the y-axis (−1.09 ± 0.62 mm) (*P* < 0.05). The changes in the x-axis (0.02 ± 0.68 mm) and z-axis (0.01 ± 0.48 mm) showed no significant difference between before and after orthognathic surgery (Table 2).

**Table 1:** Changes in coordinate values of each patient’s preoperative and postoperative condylar heads

Patient number	Age/sex	Surgery type	Change in coordinate values of left condylar head after orthognathic surgery			Change in coordinate values of right condylar head after orthognathic surgery		
			*x*	*y*	*z*	*x*	*y*	*z*
Patient 1	29/M	Mandibular surgery	0.52	−0.58	0.81	−0.21	−1.91	−0.32
Patient 2	20/F	Bimaxillary surgery	0.02	−0.89	0.14	−0.63	−0.48	0.38
Patient 3	24/M	Bimaxillary surgery	0.36	−1.37	−0.13	0.45	−1.33	−0.26
Patient 4	24/F	Bimaxillary surgery	−0.55	−1.46	−0.66	1.08	−2.29	−0.10
Patient 5	24/M	Bimaxillary surgery	−0.03	−0.30	0.88	0.96	0.08	0.23
Patient 6	20/F	Bimaxillary surgery	0.38	−1.52	0.01	−0.22	−1.76	1.06
Patient 7	22/F	Bimaxillary surgery	−0.11	−2.00	0.03	0.36	−0.94	−0.40
Patient 8	25/F	Bimaxillary surgery	1.78	−0.98	−0.46	−0.72	−1.59	−0.70
Patient 9	24/M	Mandibular surgery	−0.24	−0.76	−0.24	−0.28	−0.53	−0.02
Patient 10	26/F	Mandibular surgery	−0.62	−1.19	−0.37	0.56	−0.44	0.24
Patient 11	18/F	Mandibular surgery	−0.69	−0.52	−0.27	−0.91	−1.14	0.41

’–‘value on the x-axis indicates right; ‘+’value on the x-axis indicates left; ‘–‘value on the y-axis indicates downward; ‘+’value on the y-axis indicates impaction; ‘–‘value on the z-axis indicates advance; ‘+’value on the z-axis indicates setback

**Table 2:** Surgical changes in condylar position after surgery, using BOS system or intended manual condylar positioning

		BOS system	Manual [25]
$\overline{x}$ (sample mean) ± *s* (sample standard deviation)	*x*	0.02 ± 0.68	−0.03 ± 3.79
	*y*	−1.09 ± 0.62*	−2.88 ± 3.10†
	z	0.01 ± 0.48	−0.51 ± 4.09
*n* (sample size)		22	36

*P < 0.05, statistically significant with paired *t*-test

†*P* < 0.05, statistically significant with ANOVA and post hoc Bonferroni technique

## Discussion

The aim of this study was to evaluate the changes in condylar positions after orthognathic surgery using virtual surgical planning via the BOS system. In this study, there was no statistically significant difference in the change in condyle after surgery in the x- and z-axes. In contrast, a statistically significant difference was observed only in the y-axis. The change in the condylar position on the y-axis after surgery occurred mainly downward, and the change was approximately 1 mm. These results are similar to those of Park et al. [25], who used the same analysis software (Table 2). Both studies used a voxel-based registration method to assess the accuracy of 3D virtually planned orthognathic surgery. Park et al. [25] determined the position of the condyle using the intended manual positioning during orthognathic surgery. A significant downward movement of the condyle was observed immediately after orthognathic surgery, but a gradual return to the preoperative condylar position was observed up to 6 months after surgery [25]. There was no statistically significant difference between the preoperative and 6-month postoperative condylar positions (a difference of less than 1 mm) [25]. Therefore, Park et al. [25] concluded that the intended manual condylar positioning might minimize changes in the condylar position. Comparatively, the changes in condylar positions after orthognathic surgery via the BOS system showed less change in the condylar position than when using the intended manual condylar positioning. Therefore, changes in the condylar positions after orthognathic surgery via the BOS system are also clinically acceptable.

Generally, three methods can be applied to assess the accuracy of 3D virtually planned orthognathic surgery: landmark-based, surface-based, and voxel-based registration, depending on the manner in which CT images are superimposed [20, 26–29]. The landmark-based registration method involves manually setting stable anatomic landmarks and superimposing them through point matching, similar to the conventional method of superimposing two-dimensional cephalometric radiographs [26]. It generates human errors, depending on the landmark setting and interobserver variations [27, 28]. The surface-based registration method involves manually setting stable anatomic regions and superimposing them by matching the corresponding closest point on the same 3D reference surface based on the interactive closest-point algorithm [26]. The voxel-based registration method is a relatively recent method used for aligning two CT images based on the grayscale differences of voxels [29]. Voxels, each with a unique grayscale value that depends on the opacity of the scanned structure, are units of volume with isotropic x, y, and z dimensions [29]. This method calculates the rotation and translation required to align two CT images based on mathematical algorithms [26]. It automatically superimposes two CT scans based on volumetric similarities and significantly reduces the possibility of human error by eliminating the need to set cephalometric landmarks multiple times [20, 28]. Although all three methods are reliable for detecting changes in landmark positions when superimposed, the surface-based and voxel-based registration methods are more accurate than the landmark-based registration method [20, 26].

A limitation of this study is that only changes in the position of the condyles immediately after orthognathic surgery were observed. The position of the condyle changes over a long period and immediately after orthognathic surgery [25]. Therefore, further studies on the long-term changes in the condylar position after orthognathic surgery using the BOS system are needed.

## Conclusion

The results of this study indicate that the changes in the condylar positions after orthognathic surgery using virtual surgical planning via the BOS system were mainly observed downward on the y-axis, with slight changes in the x- and z-axes. The change in the condylar position after orthognathic surgery using the BOS system is clinically acceptable.

## Data Availability

All data were shown in this manuscript.

## Abbreviations

BOS: Balanced orthognathic surgery
CAD/CAM: Computer-aided design/computer-aided manufacturing
RP: Rapid prototyping
3D: Three-dimensional
CPD: Condylar positioning device
CT: Computed tomography
FH: Frankfurt horizontal
DICOM: Digital Imaging and Communications in Medicine
STO: Surgical treatment objective
